# The Pocket Anatomist

**Published:** 1848-01

**Authors:** 


*The Pocket Anatomist:* being a summary description of the muscles with a tabular view of the arteries and nerves. Concordant with the Dublin Dissector and Harrison on the arteries. Buffalo; Derby and Hewson, Medical Booksellers. 1848; 16mo. p.p. 61.

We take pleasure in chronicling the publication of a Buffalo Medical work, albeit it is of rather small dimensions—a kind of bibliographical Tom Thumb, but not deficient in the qualities which belong to children of a larger growth. The object and scope of the work are fully set forth in the title page. It costs but a trifle, and every medical student will find it highly useful.

